# Microbes

**Published:** 1887-01

**Authors:** 


### Microbes.

Since Pasteur brought microbes into prominence, these little creatures have risen vastly in importance, and a new scientific branch of investigation has been instituted called "Atmospheric Micrography." An observatory has been established near Paris, and an ingenious species of trap, called *bacterimetre*, is kept continually set, on the premises, to catch them. A cubic metre of air in the observatory is found to contain on an average 530 microbes, while a cubic metre of air in the central part of the city contains nearly 10,000. According to M. de Parville, the proportion of bacteria in a cubic metre is 6 in sea air, 1 in the air of high mountains, 60 in the principal cabin of a ship at sea, 200 in the air at the top of the Pantheon in Paris, 360 in the Rue de Rivoli of Paris, 6,000 in the Parisian sewers, 36,000 in the old Parisian houses, 40,000 in the new hospital of the Hotel Dieu of Paris, and 79,000 in the old hospital of Pitie of Paris.

Wolfhugel has examined the portable waters of Berlin for bacteria. The waters of the Agnaedur from Stranlan contained in 1cc. from 1,000 to 5,000 bacteria; after filtering, however, only from 20 to 70. That from Tegel showed in 1cc. from 106 to 113 bacteria; after filtering, only from 20 to 35. The pump or ground water of Berlin was found to contain many more bacteria, the number of which amounted in one case in 1cc. to about 11,960 individuals.

It has been estimated that a single cubic inch of space will contain 8,000,000,000,000 of these little fellows, of average size. They also increase, by fission, at the rate of one division every hour; hence, a single one becomes the immediate parent of 16,777,215 every twenty-four hours, and the causative parent of 32,307,756 individuals in that length of time. Now, as each one of these 32,000,000 will become, during the next twenty-four hours, the begetter of 32,307,756 individuals, one can get a reasonable understanding of the virulency of some of our infectious diseases where there is a specific bacterium. In the space of a week or ten days, were there nothing to stay their development, they would increase to such vast numbers as to fill the bed of Lake Erie or Huron. Mathematics staggers at their enumeration.—*Pacific Record.*

